# Identification and Fine Mapping of a MYB-like Transcription Factor Regulating Yellow Grain in *Sorghum bicolor*

**DOI:** 10.3390/plants15152377

**Published:** 2026-08-03

**Authors:** Jinhong Li, Zhixin Gao, Yanpeng Zhang, Yuche Zhao, Yiwei Wang, Chunyu Wang, Shuang Gang, Xiaochun Lu

**Affiliations:** 1 Seed Industry Innovation Research Institute, Liaoning Academy of Agricultural Sciences, Shenyang 110161, China; 2 College of Agronomy, Henan Agricultural University, Zhengzhou 450046, China; 3 School of Life Science and Engineering, Shenyang University, Shenyang 110044, China; 4 International Black Soil Carbon and Sustainable Land Management Research Centre, Shenyang University, Shenyang 110044, China

**Keywords:** *Sorghum bicolor*, grain color, fine mapping, candidate genes

## Abstract

Sorghum is an important raw material for China’s baijiu (liquor) brewing industry, and the tannin content in its grains directly affects the quality and nutritional value of the liquor. Grain color is closely associated with tannin content, yet the genetic mechanism underlying grain color formation remains incompletely understood. In this study, we systematically observed grain development in the yellow-grained sorghum line GW605. The results showed that from 1 to 16 days after flowering (DAF), the grains underwent a rapid growth phase, after which the expansion rate slowed. On 25 DAF, the green color of the grains gradually faded, shifting from white to yellow and continuously deepening, ultimately turning dark yellow. A segregating population was subsequently constructed using GW605 (yellow grain) and 6E16 (white grain) as parents. Bulked segregant analysis (BSA) localized the gene controlling the yellow grain to chromosome 1. Through fine mapping based on recombinant individuals, the gene was narrowed to a 108 kb physical interval containing seven candidate genes. Sequencing and functional annotation of the candidate genes suggested that *Yellow Seed 2* (Sobic.001G397900) is the candidate gene associated with yellow grain color difference between the two parental lines. Haplotype analysis of *Yellow Seed 2* using resequencing data from 698 sorghum germplasm accessions detected a total of 11 SNP loci, forming four haplotypes, among which Hap1 was the predominant haplotype. Further analysis revealed that the haplotype diversity of *Yellow Seed 2* increased considerably during the domestication process from Chinese landraces to Chinese cultivars, suggesting that this gene may have incorporated genetic lineages from foreign germplasm during domestication.

## 1. Introduction

Sorghum (*Sorghum bicolor* L. Moench, 2n = 2x = 20) is the fifth largest cereal crop globally, trailing only maize, wheat, rice, and barley. Sorghum is widely used as a resource for food, feed, brewing, and bioenergy raw materials. In China, the principal production areas of sorghum are situated in North China and Southwest China, where over 80% of sorghum grains are used in brewing [1,2,3]. Indeed, most of the popular Chinese liquor brands use sorghum as the main raw material for brewing, including Fenjiu, Luzhou Laojiao, Wuliangye, and Moutai [4]. Among the physical and biochemical characteristics related to liquor quality, the tannin content in sorghum grains plays an important role in brewing liquors with different fragrances [5,6,7].

Grain color is generally correlated with the tannin content in sorghum, in which a darker grain color indicates a higher tannin content [8]. However, sorghum grain color is regulated by multiple factors [9,10], including the exocarp pigmentation, mesocarp thickness, and presence/absence of the testa [11]. Condensed tannins in the sorghum pericarp, also known as proanthocyanidins, are flavonoid compounds derived from secondary metabolism in higher plants [12,13]. In addition to condensed tannins, the pericarp of sorghum grains contains phenolic compounds such as phenolic acids and flavonoids, which contribute to the different colors of sorghum grains. The classic genetic model suggests that grain color and the seed coat pigment are controlled by multiple loci [14,15,16]. Specifically, the pericarp color of sorghum grains is determined by the interaction between the *R* (controlling red grains) and *Y* (controlling yellow grains) genes, leading to a variety of sorghum grain colors such as red, orange, and white, corresponding to the genotypes *R_Y_*, *rrY_*, and *R_yy* (or *rryy*), respectively.

B1 and B2 are two loci that regulate the synthesis of tannins in the seed coat (SC). Due to the presence of the *S* gene, in sorghum grains with the *B1_B2_ ss* genotype, only the SC contains tannins. In contrast, in grains with the *B1_B2_S_* genotype, the SC and the pericarp contain tannins, which typically results in a brown color of the pericarp. In addition, other regulatory genes, including *I* and *Z*, can modify the basal pericarp color of sorghum grains, collectively contributing to the wide color spectrum ranging from white to black [17]. Additionally, *Yellow Seed 1* (*YS1*; Sobic.001G398100) has been cloned, which encodes a MYB transcription factor that controls pericarp pigmentation and 3-deoxyanthocyanidins accumulation [18,19,20,21].

Zhang et al. [22] performed a genome-wide association study and employed four models (BLINK, FarmCPU, GLM, and MLM) to identify the genetic basis underlying the natural variation in sorghum grain color. They identified a total of 710 quantitative trait loci (QTLs) associated with exocarp color (EC), mesocarp color (MC), endocarp color (EN), and tannin content on all chromosomes except for chromosome 5. Among these, 47 novel and important QTLs were identified. These important QTL sites were found to be related to flavonoid biosynthesis pathways [22].

The haplotype refers to the combination of alleles at multiple loci that are inherited together on the same chromosome. Mining superior allelic variations within genetically diverse natural populations has become a core molecular breeding strategy for major economic crops, including rice, wheat, soybean, and foxtail millet. For example, the discovery of favorable haplotypes for the rice grain-width gene *GS5* provided the foundation for breeding strategies to improve rice yield and quality [23,24,25]. In addition, the wheat (*Triticum aestivum*) grain-weight gene *Grain Weight 2-6A* (*TaGW2-6A*) exhibits interactions between allelic variations in the coding and promoter regions. The T/A haplotype, as an elite allelic combination, more effectively increases wheat grain width and weight compared with the T/G, -/A, and -/G haplotypes [26,27]. Additionally, the development of markers for soybean cyst nematode resistance loci *Resistance to Heterodera glycines 1* (*Rhg1*) and *Resistance to Heterodera glycines 4* (*Rhg4*) has provided a valuable reference for selecting resistant germplasm materials [28,29]. *Glycine max Increased Leaf Petiole Angle 1* (*GmILPA1*) is an important gene controlling petiole angle in soybean. A total of 13 single-nucleotide polymorphisms (SNPs) and 19 insertion-deletion (Indel) loci were detected in 783 germplasm resources. Among them, Hap1 was the predominant haplotype and Hap3 contained two non-synonymous mutations. The numbers of polymorphic loci and haplotype distribution types of *GmILPA1* gradually decreased from landraces to cultivated varieties, indicating that beneficial variations were fixed through positive selection during domestication, exhibiting a bottleneck effect [30]. *Setaria italica Timing of CAB Expression 1* (*SiTOC1*), a gene controlling heading time in foxtail millet, exhibits rich polymorphism among different varieties. The SNP at 591 bp in the promoter region is the major-effect locus causing differences in heading time. Haplotype Hp-591A, which is an earlier-maturing haplotype than Hp-591C, can serve as a major-effect haplotype for molecular breeding selection [31,32,33].

Three haplotypes of *YS1* were identified in 242 Chinese sorghum germplasm resources: Hap1, Hap2, and Hap3. Grains with Hap1 and Hap3 exhibit a red or brown pericarp, whereas varieties with Hap2 show a white or yellow pericarp [22]. Pan-genome analysis identified a 3216 bp fragment deletion in *YS1* of the reference genome BTX623, leading to the loss of function of *YS1* [19,20,34,35,36,37,38]. However, these reported haplotype variations and functional deletions of *YS1* cannot account for the grain color difference between our two parental materials GW605 and 6E16. Genomic sequencing in the present study verified that both parents carry the identical 3216 bp deletion within the *YS1* coding region, which completely abolishes the transcriptional function of *YS1* in both lines. Consequently, the yellow-and-white grain phenotypic segregation observed in our F_2_ population is independent of *YS1* genotype, implying that an uncharacterized novel regulator dominates the yellow grain trait in this genetic background. Up to now, few genetic studies have focused on the molecular basis of pale yellow pericarp in brewing-specific sorghum germplasm, and no candidate gene responsible for this unique yellow grain phenotype has been finely mapped or characterized.

To gain a better understanding of the genetic basis of sorghum grain color, in this study, we analyzed the color changes in the yellow-grain material GW605 at different developmental stages. We further performed fine mapping of the yellow-grain trait using an F_2_ mapping population, and the target gene was ultimately mapped between the BR13 and P2 markers. Genes within the candidate interval were screened to identify *Yellow Seed 2* (*YS2*; Sobic.001G397900) as a novel gene regulating sorghum grain color. Additionally, the pericarp layer structure of mature grains with different colors was observed using a stereomicroscope, and haplotype analysis of *YS2* was conducted in 698 sorghum germplasm resources. This research provides a novel gene for breeding new brewing varieties in China and lays a theoretical foundation for further genetic studies on sorghum grain color.

## 2. Materials and Methods

### 2.1. Plant Materials

The parental materials, 6E16 (white-grain variety) and GW605 (yellow-grain variety), were derived from a natural sorghum population that is self-pollinated and has been preserved by our research group for several consecutive years. After bagging and self-pollinating the F_1_ plants, a total of 3215 individual plants were obtained in the F_2_ genetic segregation population. In 2023, the parental lines and F_2_ segregation population were planted at the experimental base of Liaoning Academy of Agricultural Sciences (42°11′51″ N, 123°25′9″ E). For resequencing and haplotype analysis, a set of 698 natural population materials were cultivated at the same experimental base consecutively from 2022 to 2024. This included 217 Chinese sorghum landraces, 122 Chinese cultivated sorghum varieties, and 359 sorghum varieties introduced from abroad. The plantings had a row length of 2 m, plant spacing of 0.25 m, and row spacing of 0.6 m, with conventional field management practices applied.

### 2.2. Analysis of Developmental Morphological Changes and Agronomic Traits in Yellow-Grained Sorghum

During the full-blooming stage, the flowering dates of GW605 plants were marked by a combination of tagging, bagging with mesh nets, and labeling with marker pens. Marking was conducted every 3 days, with five plants marked each time. Sampling for observation began 1 day after flowering (DAF) and subsequent samples were taken every 3 days until 43 DAF when the grain color stabilized. Samples were observed under a stereomicroscope (Nikon ECLIPSE 80i, Tokyo, Japan). After grain maturation of white-grain and yellow-grain sorghum materials, agronomic traits were measured, including seed length, seed thickness, spike length, spike thickness, number of first-class branches, number of spikelets, seed setting rate, and 1000-grain weight. All phenotypic evaluations of grain color and agronomic traits in this study were performed with three independent biological replicates and consistent field management conditions to ensure phenotypic accuracy and repeatability. For each replicate, more than 30 individual plants were randomly selected for phenotypic investigation throughout the grain developmental stage. Data analysis was performed using GraphPad Prism 10.2 software.

### 2.3. Mapping the Gene Controlling the Yellow-Grain Sorghum Trait

For bulked segregant analysis, two extreme phenotypic pools were constructed based on stable grain color phenotypes of F_2_ individuals. Specifically, 50 plants with pure white grains consistent with the female parent 6E16 were randomly selected to form the wild-type pool, and 50 plants with stable yellow grains consistent with the male parent GW605 were selected to construct the mutant pool. Young leaf tissues of each individual were sampled for genomic DNA extraction. The DNA quality and concentration were detected by 1% agarose gel electrophoresis and NanoDrop spectrophotometer to ensure qualified sequencing samples.

Whole-genome resequencing was performed on the Illumina NovaSeq platform. The average sequencing depth reached 36× for the wild-type pool and 36× for the yellow-grain mutant pool, with an effective genome coverage of more than 95% for each pool. Raw reads were filtered using fastp (version 1.3.6 software to remove low-quality reads, adapter sequences, and ambiguous bases. The filtering criteria were set as follows: reads with Q20 quality below 90% and N base ratio greater than 5% were eliminated.

ΔSNP index = SNP index (Mut) – SNP index(WT)

Herein, Mut and WT refer to the mutant pool and wild-type pool of the progeny, respectively. ρX and ρx represent the read counts of the allele from the wild-type parent and the allele from the mutant parent in each pool, respectively. Therefore, the final ΔSNP index can be used to detect differences at each locus between the mutant and wild-type pools.

Genomic DNA was fragmented by mechanical shearing, followed by purification, end repair, adenylation, and adapter ligation. After agarose gel electrophoresis, size selection and polymerase chain reaction (PCR) enrichment were performed to construct the sequencing library. Library quality control was conducted, and qualified libraries were sequenced using the Illumina platform. These steps enabled the initial mapping of *YS2*, the gene controlling the light-yellow grain trait.

Using the genome sequence published on Phytozome (https://phytozome-next.jgi.doe.gov/, accessed on 24 June 2026) as the reference genome, simple sequence repeat (SSR), Indel, and SNP molecular markers were developed based on the initial mapping results for the target interval. Polymorphic markers between parents were screened, and the selected markers were used for fine mapping of 3215 individual plants in the F_2_ population. Prior to variant calling, the following pre-processing steps were performed using Picard v2.27.1 and GATK v4.2.6.1: (1) removal of duplicate sequences (Mark Duplicates); (2) local realignment to correct InDel-related alignment errors; and (3) base recalibration to ensure the accuracy of SNP/InDel detection.

The PCR system comprised 0.5 μL of DNA, 5 μL of 2 × PCR premix, 4 μL of ddH_2_O, 0.25 μL forward primer, and 0.25 μL reverse primer (Supplementary Table S1). The PCR program was as follows: pre-denaturation at 95 °C for 5 min; 35 cycles of denaturation at 94 °C for 30 s, annealing at 55 °C for 30 s, and extension at 72 °C for 30 s; and a final extension at 72 °C for 10 min. PCR products were separated by 8% polyacrylamide gel electrophoresis, followed by silver staining, development, photography, and statistical analysis of recombinant plants.

### 2.4. Candidate Gene Prediction

Candidate interval genes were queried using EnsemblPlants tools (http://plants.ensembl.org/Sorghum_bicolor/Info/Index, accessed on 24 June 2026), and their functions were preliminarily predicted based on gene annotations. All genes within the candidate interval were sequenced to analyze sequence differences between parents and predicted candidate genes. The PCR system comprised 1 μL of DNA, 10 μL of KOD One™ Master Mix, 1.5 μL each of forward and reverse primers, and ddH_2_O to reach a total volume of 20 μL (Supplementary Table S2). The PCR program was as follows: pre-denaturation at 94 °C for 2 min; 30 cycles of denaturation at 98 °C for 10 s, annealing at 59 °C for 20 s, and extension at 68 °C for 10 s; and final extension at 72 °C for 4 min. PCR products were separated by 1.2% agarose gel electrophoresis and target fragments were recovered using a TaKaRa Gel Extraction Kit. The purified products were sent to Tianjin AscentBio Biotechnology Co., Ltd. (Tianjin, China) for Sanger sequencing, followed by sequence analysis.

### 2.5. Validation with Quantitative PCR (qPCR) and Phylogenetic Tree Construction

Primers were designed based on the coding sequence of *YS2* and the reference gene *SbActin*, as follows: Yellow Seed F1, CAACTACATTGCGGAGCACG; Yellow Seed R1, AGATGTTGCCCCTCTTGACG; SbActin F1, ATGGCTGACGCCGAGGATATCCA; SbActin R1, GAGCCACACGGAGCTCGTTGTAG. All primers were synthesized by Nanjing GenScript Biotech Co., Ltd. (Nanjing, China). Total RNA was extracted using TRIzol reagent, and cDNA was synthesized using the TaKaRa PrimeScript RT reagent Kit with gDNA Eraser. qPCR was performed using the TaKaRa Power SYBR Green PCR Master Mix. The reaction system comprised 0.5 μL of cDNA, 5 μL of SYBR Green PCR Master Mix, 4.1 μL of ddH_2_O, and 0.2 μL each of forward and reverse primers. The qPCR program was as follows: pre-denaturation at 95 °C for 1 min; 50 cycles of denaturation at 95 °C for 5 s, annealing at 58 °C for 25 s, and extension at 72 °C for 18 s; and final extension at 72 °C for 10 min. Each sample was subjected to three biological replicates. Relative gene expression was analyzed using the 2^−ΔΔCt^ method, and results were visualized with GraphPad Prism 12.0 software.

Amino acid sequences of *Sorghum bicolor* (sorghum), *Zea mays* (maize), *Oryza sativa* (rice), *Setaria viridis* (green foxtail), *Miscanthus floridulus* (elephant grass), *Brachypodium distachyon* (purple false brome), *Triticum aestivum* (wheat), and *Lolium multiflorum* (Italian ryegrass) were aligned using ClustalX 2.0 software. A phylogenetic tree was constructed using the neighbor-joining method in MEGA 8.0 software with a bootstrap value of 1000.

### 2.6. Resequencing of 698 Natural Population Resources

Genomic DNA was extracted from the young leaves of 698 sorghum natural population materials. Purified DNA samples were used to construct sequencing libraries using the TruSeq Nano DNA HT Sample Preparation Kit (Illumina, San Diego, CA, USA). Library samples were sent to Beijing Biomarker Technologies for high-throughput sequencing. Base calling was performed using Illumina Casava 1.8 and genome sequencing was conducted with a paired-end 150 bp sequencing strategy. The sorghum reference genome was sourced from Gene Report: Sobic.001G397900: Phytozome (doe.gov). Genotype data were filtered using PLINK v1.9 software with the following parameters: --geno 0.05 (removing SNPs with >5% missing data) and --maf 0.05 (excluding SNPs with minor allele frequency <5%). Subsequent genotype imputation was performed using BEAGLE (version 5.5_27Feb25.75f) software.

### 2.7. Haplotype Analysis

The genomic sequence of *YS2* was retrieved from Phytozome (). SNP calling was then performed using PilotEdit software v15.0. Excel was used for further filtering of SNP variants, statistical analysis of polymorphic sites, and haplotype inference. Subsequently, haplotype analysis was performed using Haploview (version 4.2, http://www.broad.mit.edu/mpg/haploview/, accessed on 24 June 2026). By calculating the linkage disequilibrium (LD) coefficients between adjacent SNP loci, adjacent SNPs within the 95% confidence interval of LD values were grouped into the same haplotype block. Based on the haplotype block patterns and phenotypic data, superior haplotype combinations were identified. Missing genotype data were imputed with the reference allele by default.

### 2.8. Observation of Pericarp Layer Structure and Determination of Tannin Content in Sorghum with Different Grain Colors

Healthy, plump sorghum grains free of pests and diseases were selected at maturity for observation. Four genotypes with distinct grain colors were chosen: Hap1 (GW760, red), Hap2 (GLB41, orange), Hap3 (GW605, yellow), and Hap4 (6E16, white). The seeds were placed on the workbench with the embryonic side upward, longitudinally sectioned with a surgical blade, and then examined under a stereomicroscope to observe the color of the EC, MC, and EN in the four grain types. Three seeds each were selected from Hap1 (GW760), Hap2 (GLB41), Hap3 (GW605), and Hap4 (6E16), and the tannin content was determined via enzyme-linked immunosorbent assay (ELISA) with the Tannin Content Assay Kit (Suzhou Mengxi Biomedical Technology Co., Ltd., Suzhou, China) according to the manufacturer instructions. The tannin content (mg/g) was calculated according to the following formula: [0.154 × (ΔA − 0.026)]/sample mass (g). The standard curve employed was y = 6.4769x + 0.026 (with R^2^ = 0.9991), where y corresponds to ΔA (ΔA = absorbance of the test tube − absorbance of the blank tube) and x represents the concentration (mg/mL) of the standard substance. The volume of the extract was 1 mL.

## 3. Results

### 3.1. Developmental Process and Agronomic Traits in Yellow-Grain Sorghum

**Figure 1:**
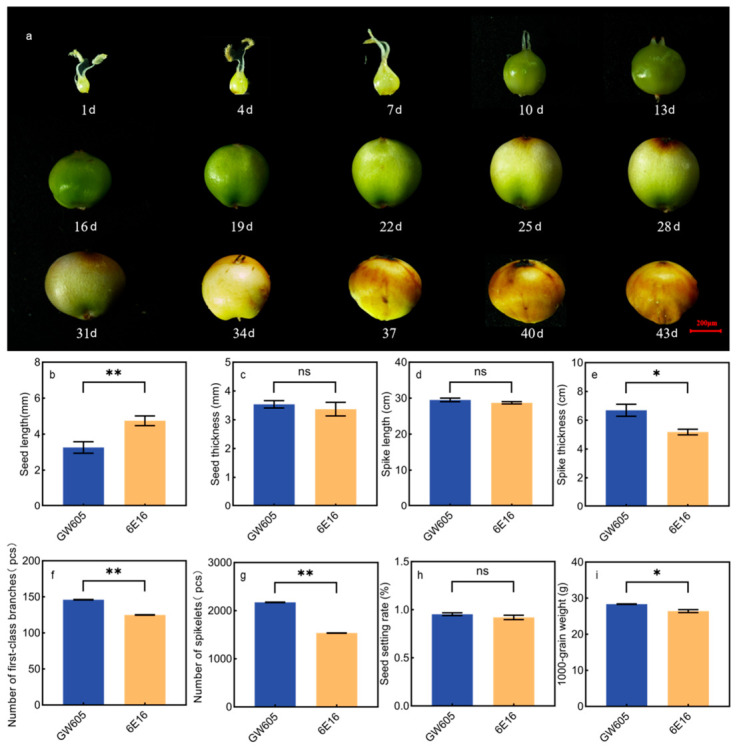
Agronomic traits of sorghum grains. (**a**) Process of sorghum grain development. (**b**) Seed length. (**c**) Seed thickness. (**d**) Spike length. (**e**) Spike thickness. (**f**) Number of first-class branches. (**g**) Number of spikelets. (**h**) Seed setting rate. (**i**) 1000-grain weight. Data are presented as mean ± standard deviation (SD). Statistical significance was analyzed by Student’s *t*-test. Asterisks indicate statistical significance: * *p* < 0.05, ** *p* < 0.01, ns represents no significant difference.

The grain development of GW605 sorghum was monitored every 3 days after flowering. From 0 to 16 DAF, the grains entered a rapid growth phase. Between 17 and 24 DAF, the grain growth rate slowed down. Starting from 25 DAF, the grains no longer expanded, which was followed by a color-transition stage in which the green color gradually faded and the grains turned milky or pale white. From 37 to 40 DAF, the grain color gradually deepened, and by 40 DAF, the grains turned yellow, which remained stable (Figure 1a).

At maturity, the panicle traits of GW605 and 6E16 were observed. GW605 had a slightly higher grain width, panicle length, and seed setting rate than 6E16, but the differences were not statistically significant. GW605 exhibited a significantly higher panicle width and 1000-grain weight than those of 6E16. Additionally, GW605 and 6E16 showed highly significant differences in grain length, number of primary branches, and number of grains per panicle (Figure 1b–i, Supplementary Table S3).

### 3.2. Mapping the YS2 Gene

To map the gene controlling the yellow-grain trait in sorghum, an F_2_ segregation population was constructed using the natural populations GW605 and 6E16. Fifty plants with the yellow-grain phenotype and 50 plants with the white-grain phenotype were selected, genomic DNA was extracted, and equal amounts were pooled to form a yellow-grain pool and a white-grain pool.

After strict quality control, the pooled data were used for subsequent analysis. After adapter trimming and low-quality read filtering of the raw data, a total of 5.0344 million clean reads and 75.52 Gb of clean bases were obtained. The core quality parameters of each sample are listed in Supplementary Table S4. Overall, Q20 ≥ 96.09% (error rate ≤ 1%), Q30 ≥ 90.03% (error rate ≤ 0.1%), and the GC content ranged from 43.99% to 45.26%. No AT/GC separation was observed, and the insert size of the library showed a normal distribution, meeting the sequencing quality requirements. The chromosome coverage depth was uniformly distributed, with only slight fluctuations in repetitive sequence regions, indicating good sequencing randomness (Supplementary Table S4).

**Figure 2:**
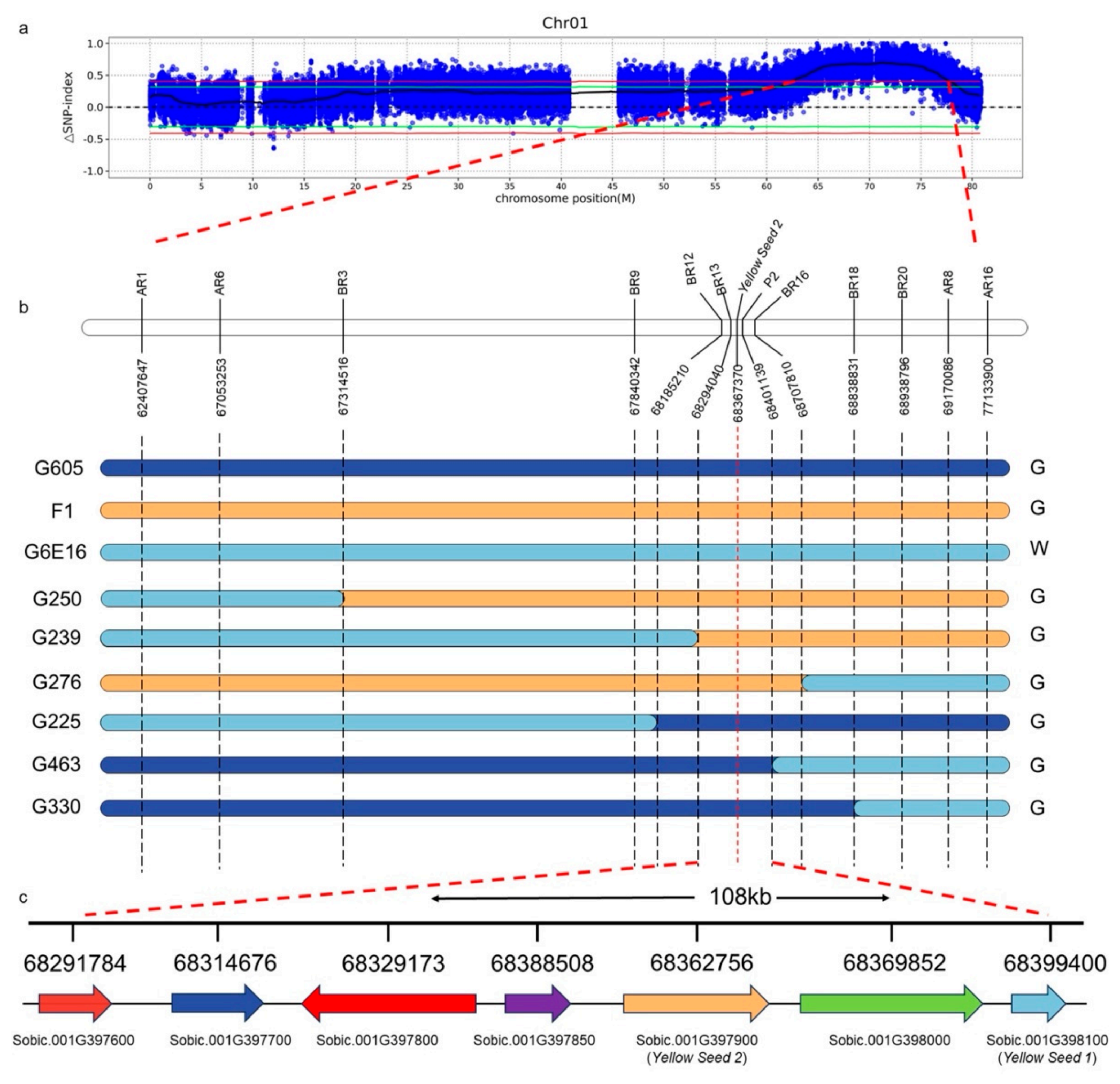
Genetic mapping of the gene controlling the light-yellow grain trait in sorghum. (**a**) Distribution of SNP-index correlation values on chromosomes. (**b**) Fine mapping delimited *YS2* to a 108 kb interval between markers BR13 and P2 on chromosome 1. “G” represents the red phenotype and “W” represents the white phenotype. (**c**) Based on the reference genome information from the sorghum gene annotation website, seven annotated candidate genes were identified within the target region. Among them, *Sobic.001G397900*. was identified as the most likely candidate gene.

According to the bulk-segregant sequencing results (Figure 2a, Supplementary Figure S1) and combined with the parental information, the △SNP index was calculated using the △SNP-index association analysis method. The calculation results were fitted, and the fitting results from computer simulation experiments exceeded the 99% threshold. As a result, the candidate gene controlling the yellow-grain trait in sorghum, *YS2*, was initially mapped to a 15.4 Mb region (62,402,294 to 77,810,226 bp) on chromosome 1.

In combination with the genomic information of multiple sequenced sorghum varieties available on the Phytozome 13 platform (https://phytozome-next.jgi.doe.gov/, accessed on 24 June 2026), we developed 8359 pairs of molecular markers (including SSRs, Indels, and SNPs) targeting the initially mapped genomic interval. Using 12 polymorphic markers (AR1, AR6, BR3, BR9, BR12, BR13, P2, BR16, BR18, BR20, AR8, and AR16), we performed fine mapping on 3215 individual plants from the F_2_ segregation population, which further narrowed down the candidate region. A total of 200 recombinant individuals were identified between markers BR12 and BR16, resulting in the initial localization of *YS2* to a 0.54 Mb interval flanked by these two markers.

To further narrow down the mapping interval, polymorphic markers between BR12 and BR16 were developed, eventually localizing the gene *YS2* controlling the yellow-grain trait to a 108 kb region flanked by markers BR13 and P2 (Figure 2b). Using gene annotation data from the sorghum genome database (http://plants.ensembl.org/Sorghum_bicolor/Info/Index, accessed on 24 June 2026), all genes and their functional annotations within the 108 kb interval were retrieved, identifying seven candidate genes. This interval contains six known protein-coding genes and one gene with unknown function (Figure 2c, Supplementary Table S5).

### 3.3. Prediction and Analysis of Candidate Genes for Yellow-Grain Sorghum

Sequencing validation was performed for the two parental lines GW605 (yellow grains) and 6E16 (white grains). Nucleotide sequences of *Sobic.001G397600*, *Sobic.001G397850*, and *Sobic.001G398000* showed no changes. *Sobic.001G397700* and *Sobic.001G397800* had five SNPs, but no amino acid sequence alterations were observed. *Sobic.001G398100* (*YS1*) was found to be associated with *MYB domain protein 11* (*MYB11*), a gene previously reported to regulate sorghum grain pericarp color [39,40]. Pan-genome analysis revealed deletions in the 5′ untranslated region (UTR) and the first two exons of this gene in four sorghum varieties, including the sequenced line BTX623, leading to functional loss [22,23].

**Figure 3:**
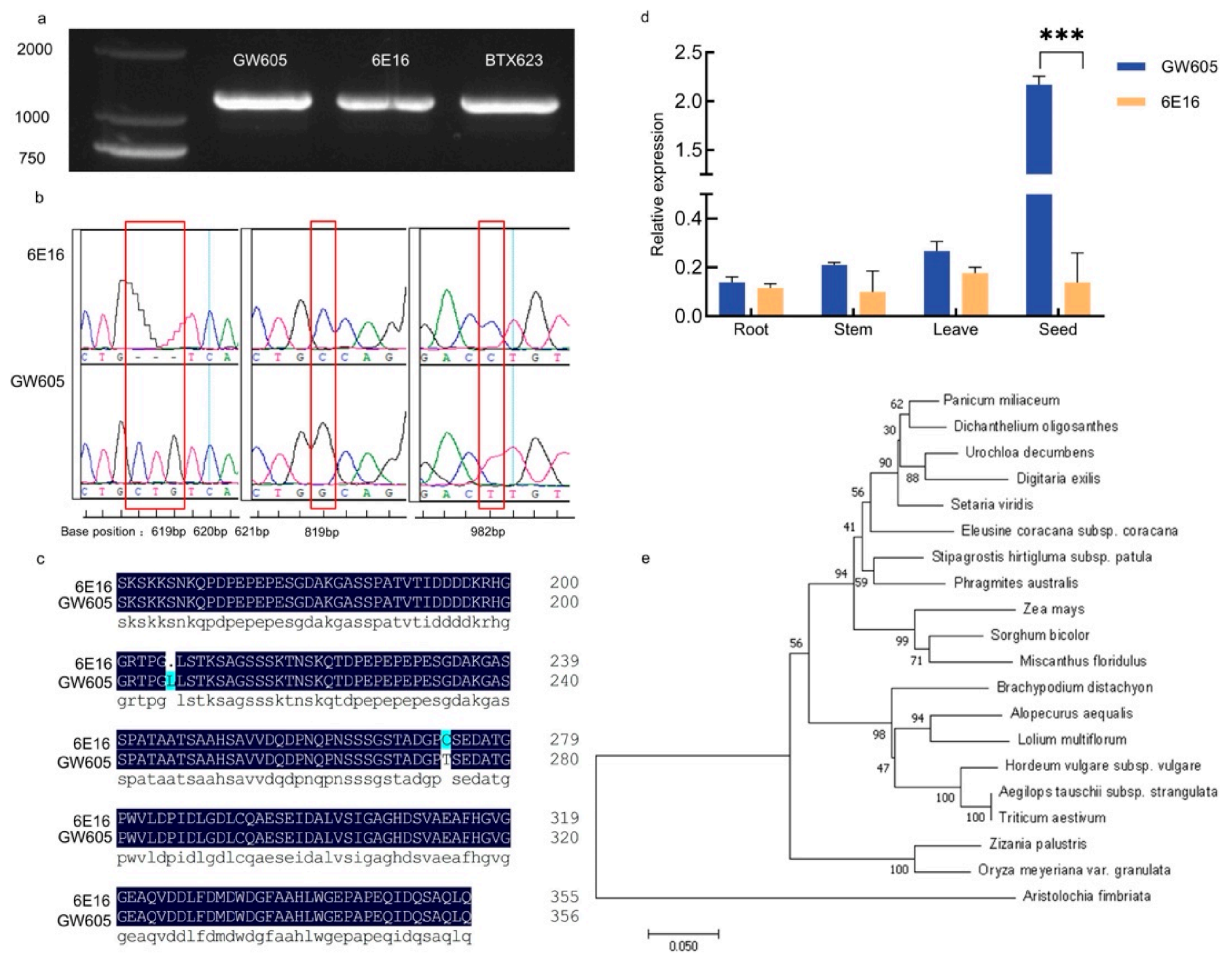
Identification, expression, and phylogenetic tree analysis of *YS2* in sorghum. (**a**) Electrophoresis detection of YS1 in GW605, 6E16, and BTX623. (**b**) Electrophoresis detection of *YS2* in GW605 and 6E16. (**c**) Amino acid sequence alignment in GW605 and 6E16. (**d**) Gene expression analysis of *YS2* with qPCR. (**e**) Phylogenetic tree of *YS2* among species. *Sorghum bicolor*: XP_002465387.1; *Zea mays*: XP_020401761.1; *Oryza* sp.: KAF0910114.1; *Setaria viridis*: XP_034571973.1; *Triticum aestivum*: XP_044375446.1; *Aristolochia fimbriata*: KAG9459592.1; *Hordeum vulgare*: XP_044981803.1; *Panicum miliaceum*: RLN42052.1; *Dichanthelium oligosanthes*: OEL35536.1; *Urochloa decumbens*: CAL4939036.1; *Digitaria exilis*: KAF8683874.1; *Eleusine*: KAK3147481.1; *Stipagrostis*: KAL6648280.1; *Phragmites australis*: XP_062215862.1; *Miscanthus floridulus*: XP_066388154.1; *Brachypodium distachyon*: XP_003558096.1; *Alopecurus aequalis*: CAM0872846.1; *Lolium multiflorum*: KAK1650754.1; *Aegilops tauschii*: XP_020199233.1; *Zizania palustris*: KAG8096289.1 Data are presented as mean ± standard deviation (SD). Statistical significance was analyzed by Student’s *t*-test. Asterisks indicate statistical significance: *** *p* < 0.001; ns represents no significant difference.

Agarose gel electrophoresis validation showed that the *YS1* gene in parental materials GW605 and 6E16, similar to the BTX623 material, had a 3.2 kb deletion, including the 5′-UTR and the first and second exons (Figure 3a, Supplementary Table S6). However, because the grains of the two parental materials exhibit yellow and white color, respectively, it was considered that the difference in grain color is not regulated by *YS1*, but by another MYB-like transcription factor in the candidate interval. As this gene appears to regulate the yellow-grain trait in sorghum, it was named *YS2*. Sequencing analysis revealed that the *YS2* coding region in GW605 (yellow grains) contains a 3 bp insertion (CTG) at position 619–621, resulting in an additional leucine residue. A non-synonymous mutation (C819G) causes a cysteine-to-tryptophan substitution, whereas a synonymous mutation (C912T) does not alter the amino acid sequence (Figure 3b,c, Supplementary Figure S2). These allelic variations strongly suggest that *YS2* is a novel gene regulating grain color in sorghum.

The *YS2* gene is located on chromosome 1 of sorghum at position 68,362,756–68,367,370, spanning a total length of 4616 bp. Its genomic structure consists of a 5′-UTR, three exons, two introns, and a 3′-UTR. The gene contains an open reading frame of 1071 bp, encoding a protein of 357 amino acids.

We next performed qPCR to analyze the tissue-specific expression of *YS2* (Figure 3d). Expression levels were calculated using the 2^−ΔΔct^ method. *YS2* was expressed in various tissues, with the highest expression level detected in the mature seeds, followed by the leaves and stems, and the lowest expression level found in the roots. Overall, the expression level of *YS2* in GW605 was significantly higher than that in 6E16. This suggests that *YS2* may positively regulate the biosynthesis of flavonoids, thereby influencing sorghum grain color variation.

Amino acid sequences of *YS2* from 20 species were aligned and a phylogenetic tree was constructed (Figure 3e). The tree clearly clustered into four distinct clades. *Sorghum bicolor* showed the closest genetic relationship with *Miscanthus floridulus*, followed by *Zea mays*, whereas *Aristolochia fimbriata* exhibited the farthest genetic distance from *Sorghum bicolor*.

### 3.4. SNP Distribution and Haplotype of YS2

**Figure 4:**
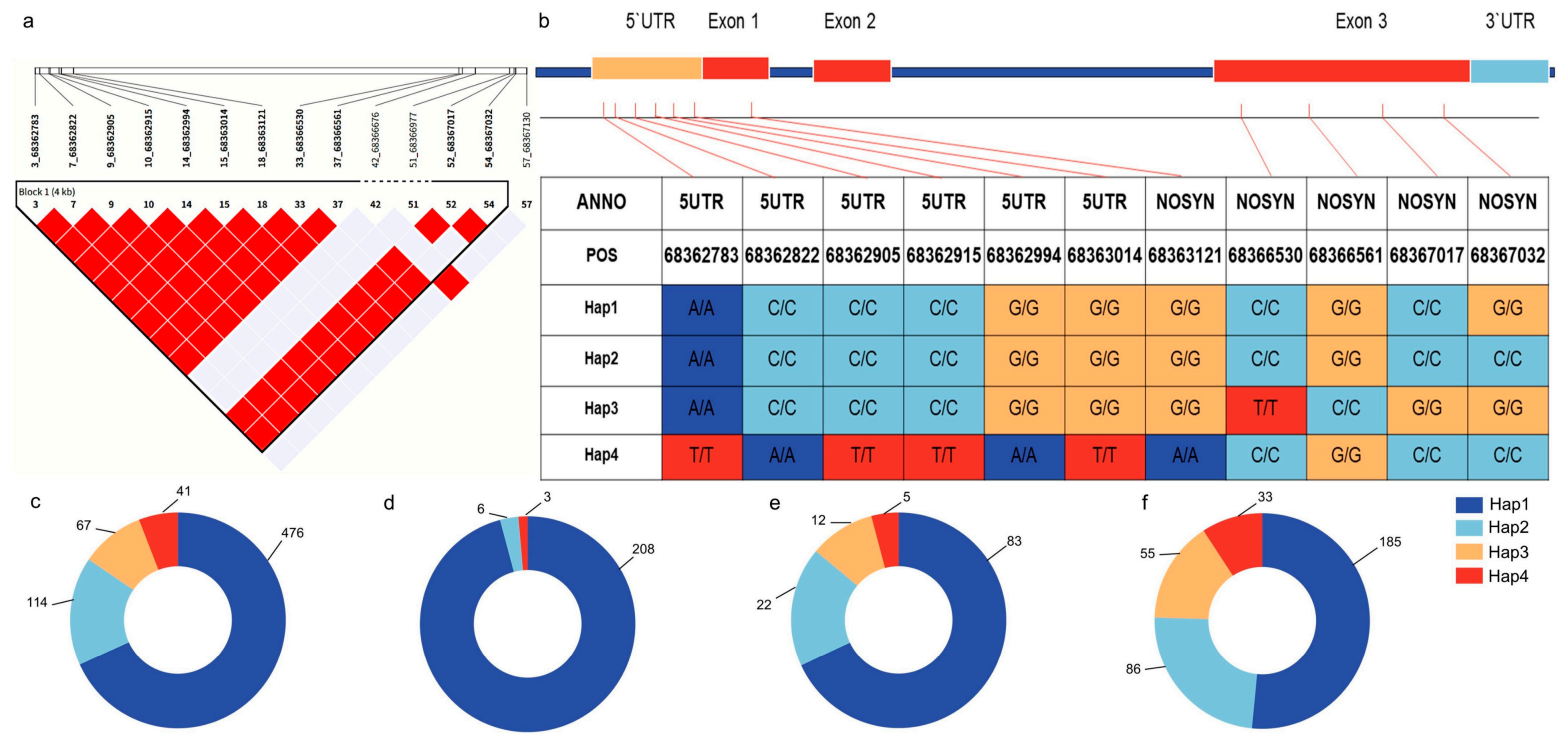
SNP distribution and haplotype analysis of *YS2*. (**a**) Linkage disequilibrium (LD) analysis of *YS2*. (**b**) Distribution of 11 SNP loci. The schematic above shows the haplotype analysis of *Sobic.001G397900*. (*YS2*), where orange and sky-blue squares denote the untranslated region (UTR) structures of the gene, red squares represent exons, and blue squares signify introns. The central red connecting lines indicate variant sites in the coding region. The information table below shows the different haplotype combinations containing 11 SNP loci and four haplotypes. In the table, distinct colors represent specific bases: blue, red, sky-blue, and orange cells denote the bases A, T, C, and G, respectively. ANNO indicates the locus annotation in the first row; 5UTR and 3UTR refer to loci in the UTRs of the gene structure; NOSYN signifies non-synonymous mutation sites; and POS denotes the physical position. (**c**) Genetic haplotype distribution map of 698 sorghum materials. (**d**) Genetic haplotype distribution map of 217 traditional farmland accessions. (**e**) Genetic haplotype distribution map of 122 cultivated sorghum accessions. (**f**) Genetic haplotype distribution map of 359 foreign sorghum materials.

To further explore the variation in the *YS2* gene in natural population materials, haplotype analysis was performed using resequencing data from 698 sorghum resources, including 217 Chinese sorghum landraces, 122 Chinese sorghum cultivars, and 359 foreign sorghum germplasm resources. The 698 sorghum natural population resources harbored 28 SNP loci (Supplementary Figures S3 and S4), including 11 variants in the coding region and 17 in the non-coding region. We conducted LD analysis across 28 loci, revealing that 14 SNPs were grouped into blocks, with 11 SNPs exhibiting LD (Figure 4a); these blocks contained four distinct haplotypes (Figure 4b). Haplotype analysis was conducted on the 11 variant sites. Hap1 was the predominant haplotype, with a frequency of 68.0%, followed by Hap2 (16.3%), whereas Hap4 showed the lowest frequency at only 5.8%. We counted the number of haplotypes of different types of sorghum in Chinese traditional farmland sorghum resources, Chinese cultivated sorghum resources, and foreign sorghum germplasm resources. In contrast to the traditional farmland materials, the 122 Chinese sorghum cultivars and the 359 foreign germplasm resources both harbored all four haplotypes (Hap1–Hap4). This indicates that Hap3 was introgressed from foreign sorghum germplasm during artificial breeding (Figure 4c–f).

### 3.5. Structural Analysis of the Pericarp Layer in Sorghum Grains

**Figure 5:**
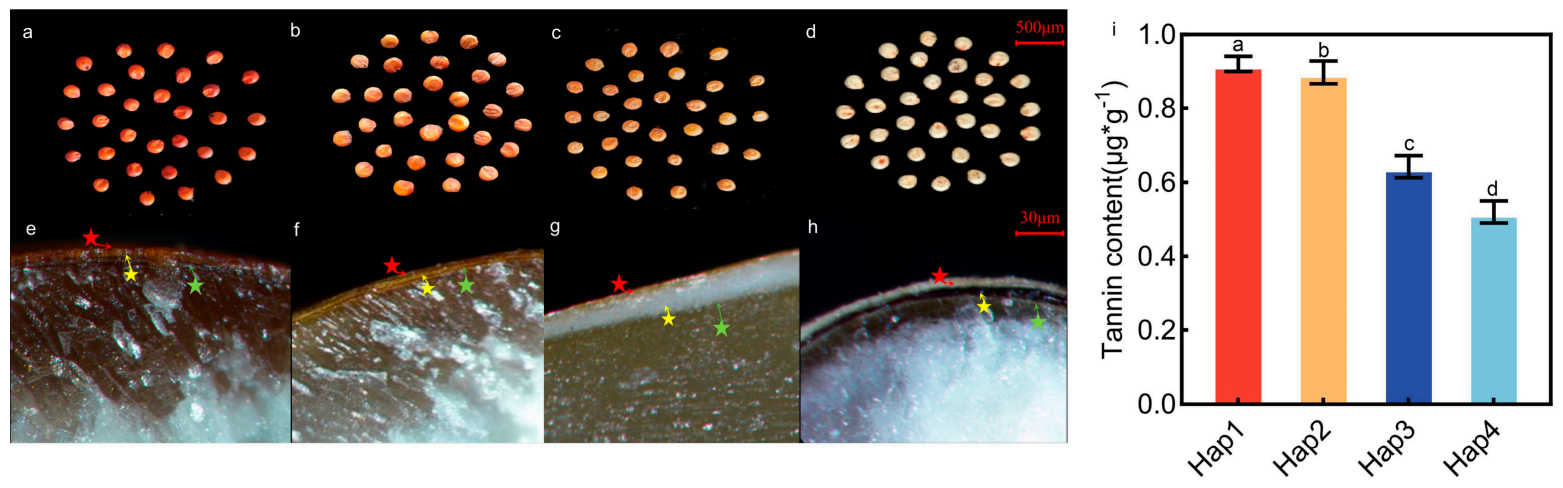
Color of the pericarp layer in sorghum grains with different kernel colors. (**a**–**d**) seed color, bar = 500 μm: (**a**) Hap1 (GW760, red grains); (**b**) Hap2 (GLB41, orange grains); (**c**) Hap3 (GW605 yellow grains); (**d**) Hap4 (6E16, white grains). (**e**–**h**) Pericarp color of the (**e**) white-grain peel structure, (**f**) yellow-grain peel structure, (**g**) orange-grain peel structure, and (**h**) red-grain peel structure, bar = 30 μm. (**i**) Tannin content. Red star: exocarp; yellow star: mesocarp; green star: endocarp. Data are presented as mean ± standard deviation (SD). Statistical significance was determined using one-way ANOVA followed by Tukey’s multiple comparison test. Different lowercase letters indicate significant differences at *p* < 0.05.

The color of sorghum grains is influenced by multiple factors, including pericarp color, MC thickness, and the presence or absence of the SC. The pericarp of sorghum grains develops from the ovary wall. The pericarp generally consists of three tissue layers: the EC, MC, and EN. The outermost layer is the EC, composed of 2–3 layers of rectangular cells. The MC is made up of several layers of large, elongated parenchyma cells, whereas the EN consists of cross cells and tube cells. We examined the pericarp structure of mature grains from four distinct sorghum haplotypes using a stereomicroscope. The EC and EN of Hap1 (GW760) were reddish-brown, the MC was brownish-black, and the endosperm tissue was also brownish-black (Figure 5a,e). This may be due to the pigments in the sorghum pericarp penetrating the endosperm tissue inside the grain through osmosis. The EC and MC of Hap2 (GLB41) were brownish-yellow, whereas the EN was brown (Figure 5b,f). The EC and EN of Hap3 (GW605) were yellow and the MC was white (Figure 5c,g). The EC and EN of Hap4 (6E16) presented as white, whereas the MC showed a deep-red color (Figure 5d,h); this latter type of sorghum is typically used as edible sorghum

Proanthocyanidins are a class of phenolic compounds that play a crucial role in Chinese liquor and wine brewing. Moderate tannin levels inhibit spoilage microbes and facilitate the production of vanillic acid, syringic acid, and eugenol, endowing alcoholic beverages with unique aromas. Thus, tannins in the pericarp are vital for the brewing process. Additionally, sorghum with a high tannin content is less susceptible to bird predation. In general, darker sorghum pericarp color is positively associated with higher tannin accumulation, which is consistent with our results showing tannin content increased with the darkening of EC color (Figure 5i). Nevertheless, pericarp color alone cannot serve as a fully reliable indicator of tannin levels in sorghum [41], and not all red or brown-pericarp sorghum varieties accumulate substantial tannins [42], indicating the existence of additional regulatory factors governing tannin synthesis.

## 4. Discussion

In this study, an F_2_ mapping population was constructed using two materials with different grain colors, 6E16 and GW605, and the gene controlling sorghum grain color traits was fine-mapped to a 108 kb interval between markers BR13 and P2 on chromosome 1. Among the seven candidate genes in this interval, *YS1* and *YS2* are annotated as MYB-type transcription factors. MYB transcription factors are widely distributed in plants and represent one of the largest transcription factor families, renowned for their diverse and essential functions in regulating the expression of numerous genes [32,33,43]. MYBs often form MBW complexes with WD40 and bHLH proteins, governing the expression of genes involved in the flavonoid biosynthetic pathway. This regulatory network thereby controls the synthesis of flavonoids, including flavones, flavanones, flavonols, and anthocyanins [21,44,45,46]. The R2R3 family of MYB transcription factors in the model plant *Arabidopsis thaliana* is divided into 25 subgroups [47,48,49]. Members of subgroups S4–S7 are involved in flavonoid biosynthesis, among which the S7 subgroup members *AtMYB12*, *AtMYB11*, and *AtMYB111* positively regulate flavonol production [50], whereas the subgroup S6 member *AtMYB75* positively controls anthocyanin synthesis [51,52]. The *Brassica rapa* (Chinese cabbage-type rapeseed) *Transparent Testa 1* (*BrTT1*) gene encodes a transcription factor regulating SC pigment biosynthesis. Sequencing of *BrTT1* in 22 rapeseed resources with different grain colors (black, reddish-brown, and yellow seeds) revealed seven SNPs in the exons and two fragment deletions in the introns of *BrTT1* from yellow-seeded rapeseed. These variations lead to changes in rapeseed grain color [53,54,55,56].

The candidate interval identified in this study contains two MYB transcription factors: *YS1* and *YS2*. Previous studies reported that *YS1* is a key gene controlling sorghum grain pericarp color [20,21,22]. Three haplotypes were identified in 242 Chinese sorghum germplasm resources, indicating that the gene sequence is relatively conserved among these resources. Sorghum varieties with haplotypes Hap1 and Hap3 exhibit a red or brown pericarp, whereas those with Hap2 show a white or yellow pericarp. Pan-genome analysis revealed that four sorghum lines, including that used to establish the reference genome (BTx623), harbor a 3.2 kb deletion. This deletion results in loss of the 5′-UTR, first exon, and second exon of the gene, leading to functional loss [19,20]. Agarose gel electrophoresis showed that both parental materials harbored the same 3.2 kb deletion as found in BTx623, yet their grains exhibited distinct colors. This suggests that the grain color of these materials is regulated by other genes within the candidate interval. Sequencing results revealed nucleotide changes at positions 619–621 and 819 in the coding region of *YS2*, leading to alterations in its amino acid sequence. These findings identify *YS2* as a novel gene regulating sorghum grain color.

Genetic polymorphisms in gene sequences contribute to species genetic diversity, among which SNPs and Indels are the most common. Some variations are retained during natural and artificial selection [57]. SNPs are the most frequent polymorphic markers in plant genomes [58]. Generally, non-coding regions exhibit less biological relevance and experience weaker selection pressure, thus showing relatively higher mutation frequencies. In contrast, coding regions show the opposite pattern [59]. This study used resequencing data from 698 natural sorghum population resources to detect SNPs in *YS2*, identifying 28 SNPs in total. Of these, 11 were in the coding region and 17 were in the non-coding region; no SNPs were detected in the 3′-UTR. The 11 coding-region SNPs formed four haplotypes, with Hap1 being predominant. Among the 217 Chinese sorghum landraces, three haplotypes (Hap1, Hap2, and Hap4) were identified. In contrast, the 122 Chinese sorghum cultivars and 361 foreign germplasm resources harbored all four haplotypes (Hap1–Hap4). This indicates that the foreign genetic backgrounds were introgressed into Chinese sorghum landraces during artificial breeding.

In the present study, haplotype analysis of *YS2* further revealed that allelic variations in this MYB transcription factor underlie the natural variation in exocarp color in sorghum germplasm. Distinct *YS2* haplotypes are significantly associated with different pericarp pigmentation intensities, which in turn correspond to divergent grain tannin levels. Combined with previous reports that *YS1*, Tannin1, and *I/Z* loci also participate in pericarp pigmentation and tannin biosynthesis, our findings suggest that *YS2* acts as an additional regulatory layer in the multi-genic regulatory network of sorghum grain quality. From a molecular breeding perspective, the favorable *YS2* haplotypes identified in this study can serve as robust functional markers for marker-assisted selection (MAS) of brewing-specific sorghum varieties. By precisely introgressing elite *YS2* alleles into adapted genetic backgrounds, breeders can fine-tune pericarp color and modulate tannin content to the optimal range required for Chinese baijiu production, thereby improving brewing quality and flavor consistency. This gene-targeted breeding strategy will accelerate the development of high-quality sorghum cultivars tailored for the liquor industry, with important practical value for both germplasm improvement and industrial application.

Given that grain color is regulated by multiple factors such as flavonoids, carotenoids, and proanthocyanidins, which play crucial roles in color determination, it remains unclear whether these compounds influence sorghum grain color by affecting haplotype composition. We will therefore conduct integrated analyses of transcriptomics, metabolomics, and haplotype effects to elucidate the regulatory mechanisms. These studies will advance efficient breeding strategies and provide critical guidance for developing new varieties tailored to China’s liquor brewing industry.

## Data Availability

All data supporting the findings of this study are available within the paper and its Supplementary Information.

## Supplementary Materials

The following supporting information can be downloaded at https://www.mdpi.com/article/10.3390/plants15152377/s1.

## Abbreviations

The following abbreviations are used in this manuscript:

DAF	Day after flowering
SC	Seed coat
EC	Exocarp color
MC	Mesocarp color
EN	Endocarp color
LD	Linkage disequilibrium
