# Supplementary material for: Identification and Fine Mapping of a MYB-like Transcription Factor Regulating Yellow Grain in Sorghum bicolor
Source: Plants (Basel). 2026 Aug 3;15(15):2377. doi: 10.3390/plants15152377 (PMC13467636; doi:10.3390/plants15152377)
Supplement: Supplementary file 1 [file plants-15-02377-s001.zip › plants-4429407-supplementary.pdf]

Supplementary Materials

Supplementary Table S1. Primer sequences for gene mapping

Position	Primer name	FORWARD PRIMERS (5′-3′)	REVERSE PRIMERS (5′-3′)
62407647	AR1	TCTAATCCGATTTGCCCC	AGCGCACAACAACAGCAT
67053253	AR6	CAGGCTCCATCCTATTGGT	TGGCTCCATGTCAACCAG
69170086	AR8	CCCGTATCCAAGATCCAAA	GCCTTAGTCTAGTCGCGGA
77133900	AR16	ACTGTGTGACCGTGACCCT	CATTGCTACTGCCGTTGG
67314516	BR3	TTCGATCCATGGGACCTT	TTGGGTACTTGCGGTCG
67840342	BR9	CTCGGCACATCATCTCCA	GCAAAAGCCGACCATTTC
68185210	BR12	TTGGGTTGTGGCTCTGG	GAGGTTGTGCAGAATGCAG
68294040	BR13	GGAGGAATTGCACCCAAG	TGGTCCTGCATTAGCACG
68707810	BR16	GCTTAGTTGAACGCCATCG	GATTGATGGGTGTCGGGT
68838831	BR18	TGTTCCTGCCTTCCTGGT	GCTGTTTCGTGTTTCCCC
68938796	BR20	CGATTGCCATTTTGCTTG	ATGTGAGGAGAGCGCGA
68401139	P2	CAAGACAGCGATGAATTGCC	ACTCCTCCACATCTACTCCC

Supplementary Table S2. Primer sequences for detecting candidate genes

Primer name	FORWARD PRIMERS (5′-3′)	REVERSE PRIMERS (5′-3′)
RT397600-1	CAACATGGATTGCATGCCCG	GCAGTTTCGTCTCGTAGGCA
RT397600-2	CGCAGAGAAGAAGTACGGAGG	GGTGGTGAGGCGTTCTCTT
RT397600-3	GCGCAGAGAAGAAGTACGGA	GCAGGTGGGCTTACGAGG
RT397600-4	TGGAGGAGCCAAGTCATCGG	GGCGTTCTCTTCAGCTTGGG
RT397700-1	GAACGCAAAACCGAAACCGA	CTCATCGCCTCGCATTTCAC
RT397700-2	ATCCAAGAACACGGAGACGG	GCAGAACACCCCCTGGTAAAA
RT397800-1	CGCGGATTATCTGGTCGAGT	CTCGTCGTCTTCCGATGGTT
RT397800-2	GCGTGCAGTAAAAGCTGGTG	AGGGAATGCCGTGACTGATG
RT397850-1	CGGTGCTGTGGACTACTACTG	CTGCGTTCGTCGTCACCATA
RT397850-2	TGCTGTGGACTACTACTGCG	TGCGTTCGTCGTCACCATAG
RT397900-1	CAACTACATTGCGGAGCACG	AGATGTTGCCCCTCTTGACG
RT397900-2	CCAACTACATTGCGGAGCAC	TTGGAGATGTTGCCCCTCTT
RT397900-3	GGACCAGATACTCGCCAACT	TTGGAGATGTTGCCCCTCTT
RT398100-1	CGAGAGTGAGATAGACGCCC	AAGCCATCCCAGTCCATGTC
RT398100-2	ATCCGAACCAGCCCAACAG	GCGTCTATCTCACTCTCGGC
RT398100-3	GCCGAGAGTGAGATAGACGC	GATGTGGTCGTTCTGCTCCG

RT397900-1	GTCGGTCCGTTACGTGGAAG	CGGAGTATTGTCGGCGTTGA
RT397900-2	ATAGGGTACTCCGTCTGGGT	ATTGTCGGCGTTGACTCTTC
RT397900-3	ACTGCGAGGAGAGGAGTAGT	CGCGCATAGCCTCCTCTATC
RT397900-4	TCCCGGCCTATACCTACACT	AGCTTGCTCATGTCGATGGT
RT397900-5	AGGCACACGATTACTACGGC	TATGTTGCACCGCTGGCTTT
RT398000-2	GAATCATCGCCGTTCCCATC	TCCAAATCGTTGAAGTCGCC
RT398000-3	AGGCGACTTCAACGATTTGG	CAAGAAACGCGAGCAACCT

Supplementary Table S3. Agronomic properties associated with sorghum grains

Agronomic trait	G605	6E16
Seed length (mm)	3.32±0.5	4.73±0.45**
Seed thickness (mm)	3.54±0.2	3.35±0.35
Spike length (cm)	29.46±0.75	28.76±0.55
Spike thickness (cm)	6.68±0.66	5.23±0.33*
Number of first-class branches (pcs)	146±0.53	125±0.56**
Number of spikelets (pcs)	2173±7.08	1535.00±5.15**
Seed setting rate (%)	0.956±0.03	0.925±0.04
1000-grain weight (g)	28.35±0.12	26.36±0.58*

Note: Data are presented as mean ± standard deviation (SD). Statistical significance was analyzed by Student's t-test. Asterisks indicate statistical significance: * $p < 0.05$, ** $p < 0.01$, *** $p < 0.001$..

Supplementary Table S4. Statistics of sample sequencing quality and depth

Sample ID	Clean reads ($\times 10^4$ reads)	Average sequencing depth	Q20 ratio	Q30 ratio	10× Coverage (proportion of bases with ≥10× coverage)	Reference genome alignment rate
6E16	6064.69	11.97×	96.41%	90.64%	60%	95.64%
BL	18383.74	36.87×	96.19%	90.18%	93%	97.44%
GLB41	7807.22	14.65×	96.09%	90.03%	71%	91.77%
HL	18088.82	36.22×	96.32%	90.39%	93%	97.45%

Supplementary Table S5. Information of genes in the candidate interval

Gene ID	Position (bp)	Function
Sobic.001G397600	68291784–68294851	Photomorphogenesis Repressor Homolog
Sobic.001G397700	68314676–68318322	TPR Domain-Containing Protein Homolog
Sobic.001G397800	68329173–68336300	DNA Topoisomerase II-Associated Protein
Sobic.001G397850	68338508–68340278	Protein of Unknown Function

Sobic.001G397900*	68362756–68367370	MYB Domain Protein 11-Related Transcriptional Coactivator
Sobic.001G398000	68369852–68377166	Root-Specific Expressed Protein
Sobic.001G398100**	68399400–68400602	MYB Domain Protein 11 Ortholog

Note: *****Yellow seed 2*, ****Yellow seed 1***

Supplementary Table S6. Primer (Y1P1) designed to detect the 3.2-kb presence-absence variation (PAV) sequence in *Yellow Seed 1*

Name	Primers	Sequence
Y1P1	Forward	TTGTACGTGCCGTAGAATGC
	Reverse	GAGGCCGCGTAGTATGATTT

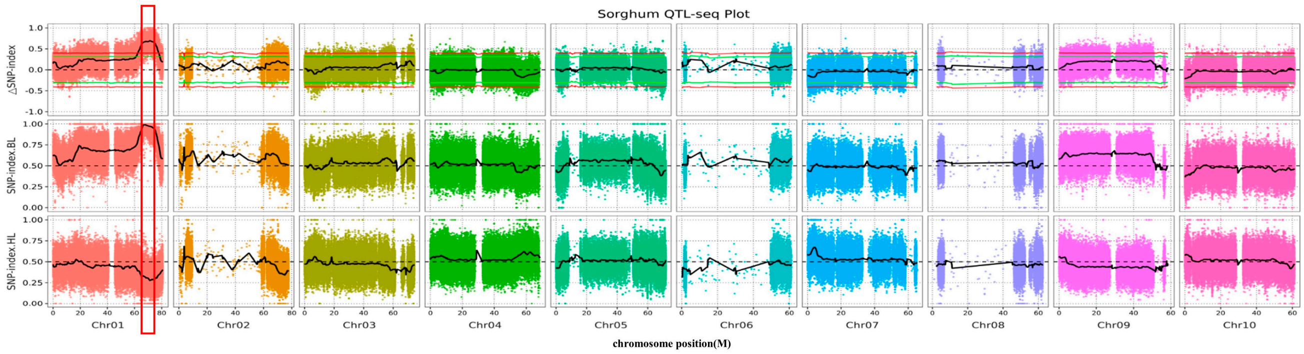

Supplementary Figure S1 Distribution of SNP-index correlation values on chromosomes

Note: The green lines indicate the 95% confidence threshold, the red lines indicate the 99% confidence threshold, and the blue box is the 15.4-Mb interval range that was initially mapped.

```
6E16.seq    NATGGGGAGGGCGCCGTGCTGCGACAAGGTGGGGCTCAAG    40
GW605.seq   NATGGGGAGGGCGCCGTGCTGCGACAAGGTGGGGCTCAAG    40
Consensus   natggggagggcgccgtgctgcgacaaggtggggctcaag

6E16.seq    CGCGGGAGGTGGACGGCGGAGGAGGACCAGATACTCGCCA    80
GW605.seq   CGCGGGAGGTGGACGGCGGAGGAGGACCAGATACTCGCCA    80
Consensus   cgcgggaggtggacggcggaggaggaccagatactcgcca

6E16.seq    ACTACATTGCGGAGCACGGCGAGGGATCCTGGAGGTCGCT   120
GW605.seq   ACTACATTGCGGAGCACGGCGAGGGATCCTGGAGGTCGCT   120
Consensus   actacattgcggagcacggcgagggatcctggaggtcgct

6E16.seq    GCCCAAGAATGCAGGGCTGCTCCGGTGCGGCAAGAGCTGC   160
GW605.seq   GCCCAAGAATGCAGGGCTGCTCCGGTGCGGCAAGAGCTGC   160
Consensus   gcccaagaatgcagggctgctccggtgcggcaagagctgc

6E16.seq    CGGCTGCGGTGGATCAACTACCTTCGGGCGGACGTCAAGA   200
GW605.seq   CGGCTGCGGTGGATCAACTACCTTCGGGCGGACGTCAAGA   200
Consensus   cggctgcggtggatcaactaccttcgggcggacgtcaaga

6E16.seq    GGGGCAACATCTCCAAGGAGGAAGAGGACATCATCGTCAA   240
GW605.seq   GGGGCAACATCTCCAAGGAGGAAGAGGACATCATCGTCAA   240
Consensus   ggggcaacatctccaaggaggaagaggacatcatcgtcaa

6E16.seq    GCTCCACGCCACCCTCGGCAACAGGTGGTCCCTGATCGCC   280
GW605.seq   GCTCCACGCCACCCTCGGCAACAGGTGGTCCCTGATCGCC   280
Consensus   gctccacgccaccctcggcaacaggtggtccctgatcgcc

6E16.seq    AGCCACCTTCCCGGCCGAACAGACAACGAGATCAAGAACC   320
GW605.seq   AGCCACCTTCCCGGCCGAACAGACAACGAGATCAAGAACC   320
Consensus   agccaccttcccggccgaacagacaacgagatcaagaacc

6E16.seq    ACTGGAACTCGCACCTCAGCCGCCAGATCCACACGTACCG   360
GW605.seq   ACTGGAACTCGCACCTCAGCCGCCAGATCCACACGTACCG   360
Consensus   actggaactcgcacctcagccgccagatccacacgtaccg

6E16.seq    CCGGAAATACACCGCCGGGCCGGACACCACCATCACCATC   400
GW605.seq   CCGGAAATACACCGCCGGGCCGGACACCACCATCACCATC   400
Consensus   ccggaaatacaccgccgggccggacaccaccatcaccatc

6E16.seq    GACATGAGCAAGCTGCAGAGCGCCGACAAGCGCCGCGGCG   440
GW605.seq   GACATGAGCAAGCTGCAGAGCGCCGACAAGCGCCGCGGCG   440
Consensus   gacatgagcaagctgcagagcgccgacaagcgccgcggcg

6E16.seq    GCAGGACTCCGGGCCGGTCGCCGAGGAGCAGCGCCAGCAG   480
GW605.seq   GCAGGACTCCGGGCCGGTCGCCGAGGAGCAGCGCCAGCAG   480
Consensus   gcaggactccgggccggtcgccgaggagcagcgccagcag

6E16.seq    CAGCAAGAGCAAGAAGAGTAATAAGCAGCCGGACCCTGAG   520
GW605.seq   CAGCAAGAGCAAGAAGAGTAATAAGCAGCCGGACCCTGAG   520
Consensus   cagcaagagcaagaagagtaataagcagccggaccctgag

6E16.seq    CCTGAACCGGAGTCCGGCGACGCGAAAGGCGCGTCCAGCC   560
GW605.seq   CCTGAACCGGAGTCCGGCGACGCGAAAGGCGCGTCCAGCC   560
Consensus   cctgaaccggagtccggcgacgcgaaaggcgcgtccagcc

6E16.seq    CGGCCACCGTCACCATCGACGACGACGACAAGCGGCACGG   600
GW605.seq   CGGCCACCGTCACCATCGACGACGACGACAAGCGGCACGG   600
Consensus   cggccaccgtcaccatcgacgacgacgacaagcggcacgg

6E16.seq    CGGCAGGACCCCGGGCCTG...TCAACGAAGAGCGCTGGC   637
GW605.seq   CGGCAGGACCCCGGGCCTGCTGTCAACGAAGAGCGCTGGC   640
Consensus   cggcaggaccccgggcctg   tcaacgaagagcgctggc

6E16.seq    AGCAGCAGCAAGACCAACAGCAAGCAGACGGACCCCGAGC   677
GW605.seq   AGCAGCAGCAAGACCAACAGCAAGCAGACGGACCCCGAGC   680
Consensus   agcagcagcaagaccaacagcaagcagacggaccccgagc

6E16.seq    CCGAGCCCGAGCCGGAGTCCGGCGACGCGAAAGGCGCGTC   717
GW605.seq   CCGAGCCCGAGCCGGAGTCCGGCGACGCGAAAGGCGCGTC   720
Consensus   ccgagcccgagccggagtccggcgacgcgaaaggcgcgtc

6E16.seq    CAGCCCGGCCACCGCCGCGACGTCGGCGGCGCACAGCGCC   757
GW605.seq   CAGCCCGGCCACCGCCGCGACGTCGGCGGCGCACAGCGCC   760
Consensus   cagcccggccaccgccgcgacgtcggcggcgcacagcgcc

6E16.seq    GTGGTGGACCAGGATCCGAACCAGCCCAACAGCAGCAGCG   797
GW605.seq   GTGGTGGACCAGGATCCGAACCAGCCCAACAGCAGCAGCG   800
Consensus   gtggtggaccaggatccgaaccagcccaacagcagcagcg

6E16.seq    GCAGCACGGCCGACGGGCCCTGCAGCGAGGACGCGACCGG   837
GW605.seq   GCAGCACGGCCGACGGGCCCTGGAGCGAGGACGCGACCGG   840
Consensus   gcagcacggccgacgggccctg agcgaggacgcgaccgg

6E16.seq    GCCGTGGGTGCTGGACCCGATAGACCTCGGGGACCTCTGC   877
GW605.seq   GCCGTGGGTGCTGGACCCGATAGACCTCGGGGACCTCTGC   880
Consensus   gccgtgggtgctggacccgatagacctcggggacctctgc

6E16.seq    CAGGCCGAGAGCGAGATAGACGCCTTGGTGTCTATCGGGG   917
GW605.seq   CAGGCCGAGAGCGAGATAGACGCCTTGGTGTCTATTGGGG   920
Consensus   caggccgagagcgagatagacgccttggtgtctat gggg

6E16.seq    CCGGCCACGATTCGGTTGCCGAAGCATTTCACGGGGTCGG   957
GW605.seq   CCGGCCACGATTCGGTTGCCGAAGCATTTCACGGGGTCGG   960
Consensus   ccggccacgattcggttgccgaagcatttcacggggtcgg

6E16.seq    TGGTGAGGCCCAGGTGGACGACCTGTTCGACATGGACTGG   997
GW605.seq   TGGTGAGGCCCAGGTGGACGACCTGTTCGACATGGACTGG  1000
Consensus   tggtgaggcccaggtggacgacctgttcgacatggactgg

6E16.seq    GACGGCTTCGCGGCCCATCTATGGGGCGAACCGGCTCCGG  1037
GW605.seq   GACGGCTTCGCGGCCCATCTATGGGGCGAACCGGCTCCGG  1040
Consensus   gacggcttcgcggcccatctatggggcgaaccggctccgg

6E16.seq    AGCAGATCGACCAGAGCGCGCAGCTGCAGCCATA        1071
GW605.seq   AGCAGATCGACCAGAGCGCGCAGCTGCAGCCATA        1074
Consensus   agcagatcgaccagagcgcgcagctgcagccata
```

Supplementary Figure S2 Coding sequence alignment of *Yellow Seed 2* between sorghum with yellow and white seeds

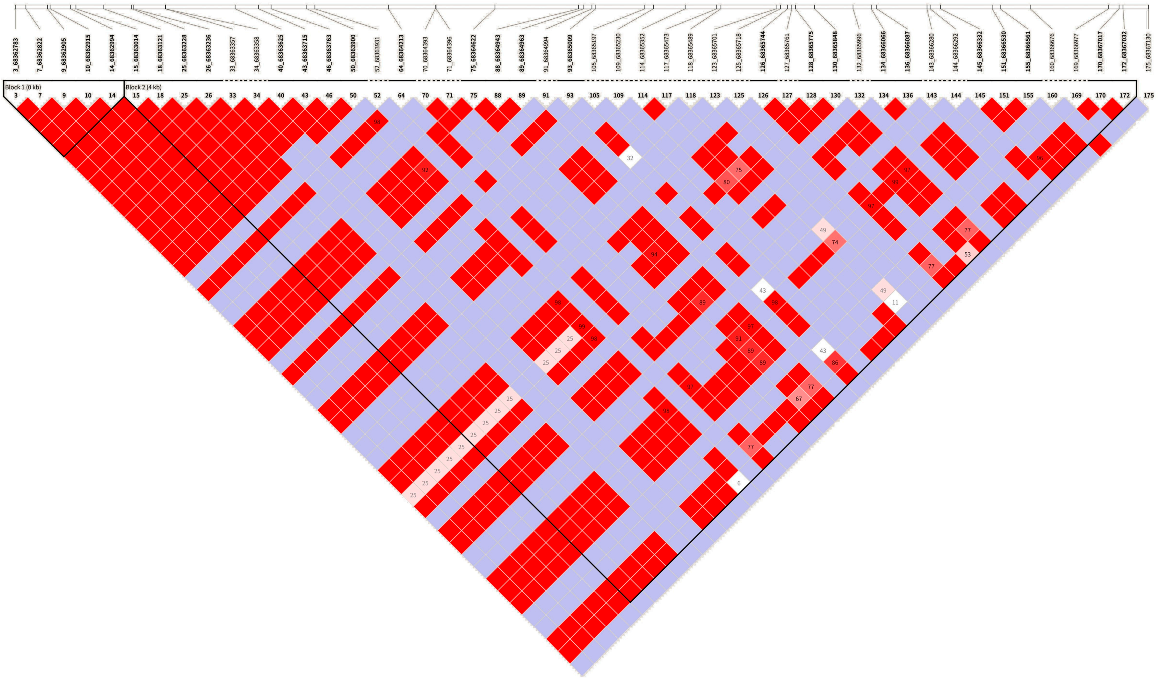

Supplementary Figure S3 Linkage disequilibrium plot of SNPs in *Yellow Seed 2*

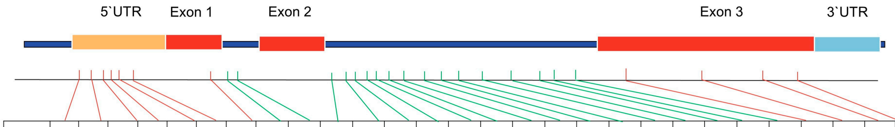

ANNO	5UTR	5UTR	5UTR	5UTR	5UTR	5UTR	NOSYN	INTRON	INTRON	INTRON	INTRON	INTRON	INTRON	INTRON	INTRON	INTRON	INTRON	INTRON	INTRON	INTRON	INTRON	INTRON	INTRON	INTRON	NOSYN	NOSYN	NOSYN	NOSYN
**POS**	68362783	68362822	68362905	68362915	68362994	68363014	68363121	68363228	68363236	68363625	68363715	68363763	68363900	68364213	68364622	68364943	68364963	68365009	68365744	68365775	68365848	68366066	68366087	68366332	68366530	68366561	68367017	68367032
**Hap1**	A/A	C/C	C/C	C/C	G/G	G/G	G/G	T/T	T/T	G/G	A/A	T/T	G/G	G/G	T/T	T/T	A/A	C/C	A/A	C/C	G/G	C/C	G/G	T/T	C/C	G/G	C/C	G/G
**Hap2**	A/A	C/C	C/C	C/C	G/G	G/G	G/G	T/T	T/T	G/G	A/A	T/T	G/G	G/G	T/T	T/T	A/A	C/C	A/A	C/C	G/G	A/A	G/G	T/T	C/C	G/G	C/C	G/G
**Hap3**	A/A	C/C	C/C	C/C	G/G	G/G	G/G	T/T	T/T	G/G	A/A	T/T	A/A	A/A	T/T	A/A	T/T	T/T	C/C	A/A	A/A	C/C	G/G	A/A	C/C	G/G	C/C	G/G
**Hap4**	A/A	C/C	C/C	C/C	G/G	G/G	G/G	T/T	T/T	G/G	A/A	T/T	A/A	A/A	T/T	A/A	T/T	C/C	C/C	A/A	A/A	C/C	G/G	A/A	C/C	G/G	C/C	G/G
**Hap5**	A/A	C/C	C/C	C/C	G/G	G/G	G/G	T/T	T/T	G/G	A/A	T/T	G/G	G/G	T/T	A/A	A/A	C/C	A/A	C/C	G/G	C/C	G/G	T/T	C/C	G/G	C/C	G/G
**Hap6**	A/A	C/C	C/C	C/C	G/G	G/G	G/G	T/T	T/T	G/G	A/A	T/T	G/G	G/G	T/T	T/T	A/A	C/C	A/A	C/C	G/G	C/C	G/G	A/A	C/C	G/G	C/C	G/G
**Hap7**	A/A	C/C	C/C	C/C	G/G	G/G	G/G	T/T	T/T	G/G	A/A	T/T	A/A	G/G	T/T	T/T	A/A	C/C	A/A	A/A	G/G	C/C	G/G	A/A	C/C	G/G	C/C	G/G
**Hap8**	A/A	C/C	C/C	C/C	G/G	G/G	G/G	T/T	T/T	G/G	A/A	T/T	G/G	A/A	T/T	A/A	A/A	C/C	C/C	A/A	A/A	C/C	G/G	A/A	C/C	G/G	C/C	G/G
**Hap9**	A/A	C/C	C/C	C/C	G/G	G/G	G/G	T/T	T/T	G/G	A/A	T/T	G/G	G/G	T/T	T/T	A/A	C/C	A/A	C/C	G/G	C/C	G/G	T/T	C/C	G/G	C/C	C/C
**Hap10**	A/A	C/C	C/C	C/C	G/G	G/G	G/G	T/T	T/T	G/G	A/A	T/T	A/A	G/G	C/C	T/T	A/A	C/C	A/A	A/A	G/G	C/C	T/T	A/A	T/T	C/C	G/G	G/G
**Hap11**	A/A	C/C	C/C	C/C	G/G	G/G	G/G	T/T	T/T	G/G	A/A	T/T	A/A	G/G	T/T	A/A	A/A	C/C	A/A	A/A	G/G	C/C	T/T	A/A	T/T	C/C	G/G	G/G
**Hap12**	T/T	A/A	T/T	T/T	A/A	T/T	A/A	C/C	A/A	A/A	T/T	G/G	A/A	G/G	T/T	T/T	A/A	C/C	A/A	C/C	G/G	C/C	G/G	T/T	C/C	G/G	C/C	C/C
**Hap13**	T/T	A/A	T/T	T/T	A/A	T/T	A/A	C/C	A/A	A/A	T/T	G/G	A/A	G/G	T/T	T/T	A/A	C/C	A/A	A/A	G/G	C/C	G/G	T/T	C/C	G/G	C/C	C/C
**Hap14**	A/A	C/C	C/C	C/C	G/G	G/G	G/G	T/T	T/T	G/G	A/A	T/T	A/A	A/A	T/T	A/A	T/T	C/C	C/C	A/A	A/A	C/C	G/G	T/T	C/C	G/G	G/G	G/G
**Hap15**	A/A	C/C	C/C	C/C	G/G	G/G	G/G	T/T	T/T	G/G	A/A	T/T	A/A	A/A	T/T	A/A	T/T	T/T	C/C	A/A	A/A	C/C	G/G	T/T	C/C	G/G	G/G	G/G

Supplementary Figure S4 SNP distribution of *Yellow Seed 2*

Note: The top figure shows the gene haplotype structure of Sobic.001G397900 (*Yellow Seed 2*), where orange and sky-blue squares denote the untranslated regions (UTRs) of the gene, red squares represent exons, and blue squares signify introns. The central red connecting lines indicate variant sites in the coding region, while the green connecting lines denote variant sites in the non-coding region. The information table below displays different haplotype combinations with 28 SNP loci. In the table, distinct colors represent specific bases: blue, red, sky-blue, and orange cells denote the bases A, T, C, and G, respectively. ANNO indicates the locus annotation in the first row; 5UTR and 3UTR refer to loci in the UTRs of the gene structure; NOSYN signifies non-synonymous mutation sites; and POS denotes the physical position.
